# Social mixing in Fiji: Who-eats-with-whom contact patterns and the implications of age and ethnic heterogeneity for disease dynamics in the Pacific Islands

**DOI:** 10.1371/journal.pone.0186911

**Published:** 2017-12-06

**Authors:** Conall H. Watson, Jeremaia Coriakula, Dung Tran Thi Ngoc, Stefan Flasche, Adam J. Kucharski, Colleen L. Lau, Nga Tran Vu Thieu, Olivier le Polain de Waroux, Kitione Rawalai, Tan Trinh Van, Mere Taufa, Stephen Baker, Eric J. Nilles, Mike Kama, W. John Edmunds

**Affiliations:** 1 Centre for the Mathematical Modelling of Infectious Diseases, London School of Hygiene & Tropical Medicine, London, United Kingdom; 2 School of Medicine, Fiji National University, Suva, Fiji; 3 The Hospital for Tropical Diseases, Wellcome Trust Major Overseas Programme, Oxford University Clinical Research Unit–Vietnam, Ho Chi Minh City, Vietnam; 4 Department of Global Health, Research School of Population Health, The Australian National University, Canberra, Australia; 5 Project H.E.A.V.E.N., Suva, Fiji; 6 Fiji Centre for Communicable Disease Control, Ministry of Health and Medical Services, Suva, Fiji; 7 Centre for Tropical Medicine and Global Health, Oxford University, Oxford, United Kingdom; 8 Faculty of Infectious and Tropical Diseases, London School of Hygiene & Tropical Medicine, London, United Kingdom; 9 Division of Pacific Technical Support, World Health Organization—Western Pacific Region, Suva, Fiji; Hokkaido University Graduate School of Medicine, JAPAN

## Abstract

Empirical data on contact patterns can inform dynamic models of infectious disease transmission. Such information has not been widely reported from Pacific islands, nor strongly multi-ethnic settings, and few attempts have been made to quantify contact patterns relevant for the spread of gastrointestinal infections. As part of enteric fever investigations, we conducted a cross-sectional survey of the general public in Fiji, finding that within the 9,650 mealtime contacts reported by 1,814 participants, there was strong like-with-like mixing by age and ethnicity, with higher contact rates amongst iTaukei than non-iTaukei Fijians. Extra-domiciliary lunchtime contacts follow these mixing patterns, indicating the overall data do not simply reflect household structures. Inter-ethnic mixing was most common amongst school-age children. Serological responses indicative of recent *Salmonella* Typhi infection were found to be associated, after adjusting for age, with increased contact rates between meal-sharing iTaukei, with no association observed for other contact groups. Animal ownership and travel within the geographical division were common. These are novel data that identify ethnicity as an important social mixing variable, and use retrospective mealtime contacts as a socially acceptable metric of relevance to enteric, contact and respiratory diseases that can be collected in a single visit to participants. Application of these data to other island settings will enable communicable disease models to incorporate locally relevant mixing patterns in parameterisation.

## Introduction

Infectious disease models synthesise epidemiological data and germ theory to understand and predict disease transmission. Non-homogeneous contact patterns are widely used in estimating the spread of an infection within a population [1,2]. For public health policy making, the prior practise of inferring social contact patterns as part of the model fitting has increasingly been replaced with data collection on social contact patterns [3,4]. This can strengthen model validity when assessing the potential impact of interventions such as school closures or vaccination [5].

Whilst social mixing has been studied in Europe [6]; Africa, including South Africa [7,8], Kenya [9], Zambia [8] and Zimbabwe [10]; Asia, including Vietnam [11], Taiwan [12], southern China [13] and Japan [14]; and Australia [15], there is a paucity of social contact data for Pacific island states. This lack of data is despite the enormous historical mortality impact of diseases such as measles and bacillary dysentery in Pacific populations [16,17], and contemporary burdens such as streptococcal diseases [18,19] and scabies [20]. Such data could also inform ongoing programmes such as trachoma elimination [21], emerging infection preparedness [22] and surveillance-response system strengthening [23], and insights from island outbreaks of pathogens such as Zika [24–26]. A sustained upturn in notified enteric fever cases caused by *Salmonella* Typhi in Fiji [27], prompted this investigation of social-mixing patterns.

Social mixing epidemiological research has predominantly considered conversational contact relevant to respiratory diseases such as influenza, or sexual contacts for infections such as HIV. Faecal-orally transmitted diseases such as typhoid are not transmitted by droplet or aerosol routes, [28,29] making conversation less relevant to transmission than mechanisms that involve food, fomites, direct contact or waterborne transmission [30]. Sexual transmission of typhoid is rare, and associated with penile-anal or oral-anal rather than vaginal sex [31]. Methods for social contact patterns estimation of relevance to enteric pathogens are required. Quantifying food-sharing contacts may be one approach [32,33].

Furthermore, whilst rarely a reported feature of social contact surveys, ethnicity–which encompasses perception of common ancestry or homeland, kinship, language, culture, physical characteristics, religion and history [34]–may also be critical to understanding disease dynamics in specific epidemiological circumstances, though requires sensitive consideration in biomedical research [35,36].

Additional to day-to-day contacts, diseases with person-to-person communicability may be spread by population movement within a country, if infection does not entirely impede mobility. A further public health threat is the spillover of disease from livestock or wildlife to humans, such as leptospirosis [37]. Zoonotic diseases may give the impression of sustained human-to-human transmission when in fact there are multiple spillover events from an epizootic [38]. Knowledge of human-animal contact patterns may inform zoonotic transmission models.

This social-mixing survey, conducted as part of a seroepidemiological survey, sought to determine 1) the distribution of social contacts by age and ethnicity 2) travel and internal migration patterns and 3) animal ownership and contact as relevant to the spread of communicable diseases in Fiji and other Pacific island settings.

## Methods

### Ethics approval

The study was approved by the Fiji National Research Ethics Review Committee (2013–03) and the London School of Hygiene & Tropical Medicine observational study research ethics committee (6344).

### Setting

Fiji is an upper-middle income state of 837,000 people in the South Pacific Ocean [39]. Administratively, Viti Levu, the largest island, is divided into Central Division (population 342,000 including the capital, Suva) and the Western Division (population 320,000). The northern Division (population 136,000) comprises the next largest two islands, Vanua Levu and Taveuni. Eastern Division (population 39,000) comprises of many smaller island groups.

An international expert meeting was convened in 2012 by the Fijian Ministry of Health and Australian Aid to investigate an upturn in typhoid fever cases from the mid-2000s. Over 90% of typhoid cases are reported in indigenous iTaukei Fijians who comprise 57% of the population,[27] giving an odds ratio >6 relative to other ethnic groups, which include Fijians of Indian descent (Indo-Fijians, 38%) and Fijians of Chinese or European descent, thus suggesting ethnicity is important in understanding transmission. Communal eating, beyond the immediate family, was commonly observed in iTaukei villages and amongst paid workers and students of both major ethnicities in Fiji. Co-dining and food-sharing was thus identified as a means of recording epidemiologically-relevant mixing patterns for enteric infections.

### Survey methods

A multistage, clustered, cross-sectional survey was done in the Central, Northern and Western Divisions of Fiji between September and December 2013 as a joint serological, risk-factor and social mixing investigation. The Eastern Division was excluded for logistical reasons, and we did not attempt to assess seasonal variation in contact patterns.

The community clusters were randomly-selected from Ministry of Health and Medical Services administrative lists for nursing zones, a contiguous health geography, with the zones selected randomly with probability proportional to population size. Within each cluster, 25 households were randomly selected. If registers were held by community health workers or nurses, these were preferentially used. Otherwise, in street-based settings, rapid enumeration of households was done with random start points and set sampling intervals. In rural villages/settlements extended program on immunization (EPI)-derived methods were used, enumerating households in random (pen-spin) directions from community centroids. One participant was randomly selected from each household. Fieldwork was done from Monday to Saturday, thereby recording social mixing for Sunday to Friday. Days for visits were determined by operational feasibility, not by randomisation and results are not reported by day. If a randomly selected household member was temporarily absent from the household at the time of the visit due to e.g. work or school, the survey team revisited later in the day after their expected return. The full survey methods have been described elsewhere [40,41] Sample size was calculated based on expected typhoid seroprevalence in 10 year age bands, the linked serosurvey’s primary endpoint. Whilst a sample size was not calculated for the social mixing survey aspect and would be inappropriate to do post-hoc, the study size is consistent with or larger than other social mixing surveys [6,11–13]. Where others report individual year contact rates we used broader age bands in the survey’s implementation and analysis to provide appropriate precision in ethnic and age strata.

The purpose of the survey was explained to community leaders if applicable, to the head of the household and the selected participant, and their permissions sought for inclusion in the survey. Written informed consent was sought and obtained from adult participants and parents of child (under 18 years) participants. Children aged 12–17 years provided written informed assent.

Interviews were done face-to-face by a trained, multilingual Fijian fieldworker in iTaukei, Hindi or English at the preference of the participant, at the participant’s home or in a community centre. Venous blood was collected by a trained phlebotomist or physician. Participants provided demographic details, including their age, sex and self-reported ethnicity. They were first asked to recall where they had lunch and dinner the previous day, to enable recording of both close extra-household and household contact rates. We then asked how many people of each age group (defined below) they ate each meal with and asked how many of the lunch and dinner contacts were the same individuals (to enable calculation of unique daily meal contacts), and asked to give their assessment of the ethnicity of co-diners. Ages of contacts were categorised into 0 to 4 years (preschool children), 5 to 14 years (school-age children), 15 to 34 years (young adults), 35 to 54 (older working-age adults) and 55+ (retirement-age adults). If more than fifteen contacts were reported in an age group, then ranges 16 to 24, 25 to 49 and 50 to 99 were recorded and midpoints of these bands used in analysis. Parents answered on behalf of young children. Infants under one were omitted from participation our study, as ineligible for the serological aspect of the field survey, though were included in the under 5s as contacts in participant responses. Participant ages were categorised as above, with the youngest band 1 to 4 years accordingly. In domestic eating settings, including villages, participants were asked to report the details of people with whom they actually shared food i.e. cooking pots or buffet meals. For those eating in settings where cooking pot sharing would be impossible to estimate and not an appropriate measure of social contact (such as at a restaurant or canteen), they were asked to report with whom they shared a table or shared a table-like setting. See supplement S1 File for the mealtime social contact questionnaire tool.

Participants were further asked about travel outside of their neighbourhood (including villages or settlements) in the past week, ever having lived in a different neighbourhood, and about animal ownership or physical (touch) contact with select wild animals.

We also sought information on physical contacts of participants. During survey piloting, candidate questions about skin-to-skin human physical contact were often met with embarrassment, and often received reports of zero contact with anyone other than between parents and infant offspring, despite observance of frequent social contact such as handshaking or arm-touching in villages and settlements. This line of enquiry was dropped to reduce participant survey fatigue and risk of social response biases in other part of the survey. Similarly, breakfast contacts were not sought due to expected overlap with dinner contacts. Fomite contacts are hard to quantify [42] and were not sought. Water-related exposures are described elsewhere [41].

### Data analysis

Data were entered in EpiData [43] and analysed in R version 3.3.2 [44]. Bootstrap 95% confidence intervals (CI) were estimated for mean contact rates. Total daily contacts between age and ethnicity subgroups were estimated based on census populations and participant-reported rates and used to construct a reciprocal-contact adjusted, symmetrical mixing matrix [45]. Binomial 95% confidence intervals were estimated for travel and animal ownership as prior analysis had found minimal influence of clustering on variances for age-structured data on similar exposures [41].

To assess possible association between mealtime contacts and biological markers of enteric infection transmission, for participants resident in unvaccinated areas, we estimated by logistic regression the age-adjusted odds ratios for iTaukei and non-iTaukei contacts and *S*. Typhi seropositivity using anti-Vi IgG titres from a linked serosurvey at thresholds 64 ELISA units (EU) and 100 EU alongside examining potential confounders. Previous research in Fiji [41] established 64 EU as the threshold towards which case titres decay; ≥100 EU is used to indicate a recent (months to a few years) infection such as may be influenced by the reported social mixing patterns.

## Results

### Study population

We received 1,814 analysable responses from 1,816 interviewees (two withdrew before contributing sufficient data, 1,842 were documented as having been approached, response rate = 98.6%). Of these, 1,409 (78%) were iTaukei ethnicity and 53.3% were female (Table 1). The median age was 30 years (IQR 17 to 48 years), with a median age of 29 years (IQR 26 to 47 years) in iTaukei and 35 years (IQR 21 to 52 years) in non-iTaukei. In comparison to the 2007 census, the non-iTaukei Fijians were under-represented amongst survey participants (supplementary S1 Fig). Over half of participants resided in rural areas, with rural living more common for the iTaukei population (60%), typically living in formal village settings (47%), than non-iTaukei (42%) who resided almost exclusively in settlements (59%) or residential housing (37%).

**Table 1:** Participant demographics.

		All participants	iTaukei	Non-iTaukei
		1,814	1,409 (78)	405 (22)
Sex (%)	Female	966 (53.3)	744 (53.3)	222 (54.8)
Age (%)	1 to 4	87 (4.8)	74 (5.3)	13 (3.2)
	5–14	299 (16.5)	246 (17.5)	53 (13.1)
	15–34	654 (36.1)	523 (37.1)	131 (32.3)
	35–54	473 (26.1)	347 (24.6)	126 (31.1)
	55+	301 (16.6)	219 (15.5)	82 (20.2)
Setting (%)	Urban	505 (27.8)	386 (27.4)	119 (29.4)
	Peri-urban	289 (15.9)	175 (12.4)	114 (28.1)
	Rural	1,020 (56.2)	848 (60.2)	172 (42.5)

### Contact patterns

**Fig 1:**
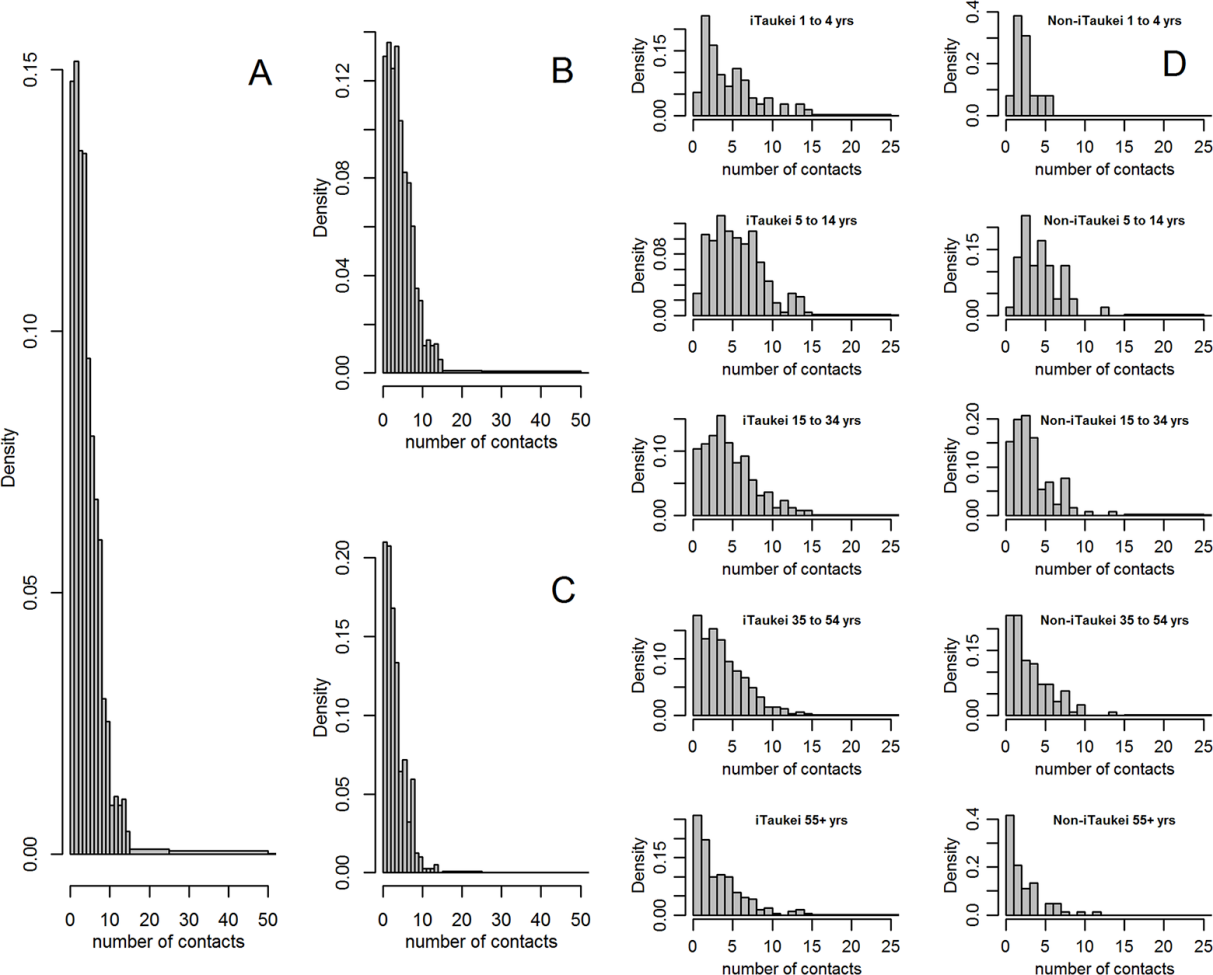
Distribution of daily contacts reported by A) all participants, B) iTaukei participants, C) non-iTaukei participants and D) participants stratified by age and ethnicity. Fig 1 A to C panels truncated at 50 contacts and D panels at 25 contacts for clarity as there were few reports of contact numbers in higher bands; densities are for the full range of reported value.

The 1,814 participants reported a total of 9,650 mealtime contacts. The distribution of daily mealtime contacts reported by participants was right-skewed (Fig 1A). Whilst both the iTaukei and non-iTaukei modal value was two (Fig 1B and 1C), the iTaukei participant’s contacts distribution had higher median (4 vs. 3) than the non-iTaukei participants and a higher interquartile range (2–7 vs. 2–5, respectively). After stratification by age and ethnicity (Fig 1D), heavy-tailed distributions were apparent for the iTaukei in comparison with equivalent-age non-iTaukei. The iTaukei aged 5 to 14 years and 15 to 34 years were the most likely to report between 5 and 10 mealtime contacts. Few respondents of any age or ethnicity reported more than ten such contacts.

Residents of Fiji exhibited strong assortative mixing by age within the two ethnicity categories (Table 2). The highest mean reported contact rate was for iTaukei participants aged between 5 to 14 years who shared a meal with people of the same age and ethnicity (3.2 contacts per day; 95% CI 2.67 to 3.98). Few contacts were reported with people of different ethnic groups to the respondents; all iTaukei participant age groups had confidence intervals that included zero for contacts of different ethnicity. The highest mean reported heteroethnic contact rates were reported by Non-iTaukei participants aged between 5 and 14 years with their iTaukei counterparts of the same age at 0.61 contacts per day (95% CI 0.25 to 1.14).

**Table 2:** Unweighted mean number of daily contacts by age and ethnicity and 95% bootstrap confidence interval.

		Contacts										
		iTaukei						Non-iTaukei				
		Age	0 to 4	5 to 14	15 to 34	35 to 54	55+	0 to 4	5 to 14	15 to 34	35 to 54	55+
**Participants**	**Non-iTaukei**	**1 to 4**	0	0.07 (0 to 0.23)	0	0.29 (0 to 0.73)	0.2 (0 to 0.62)	0.13 (0 to 0.5)	0.29 (0 to 0.67)	0.77 (0.26 to 1.3)	0.54 (0.2 to 1)	0.45 (0.09 to 0.93)
		**5 to 14**	0.09 (0.02 to 0.2)	0.61 (0.25 to 1.14)	0.18 (0.06 to 0.34)	0.31 (0.06 to 0.8)	0.02 (0 to 0.07)	0.17 (0.07 to 0.27)	1.56 (1.09 to 2.15)	0.75 (0.42 to 1.31)	0.93 (0.68 to 1.19)	0.33 (0.18 to 0.53)
		**15 to 34**	0.16 (0.06 to 0.31)	0.13 (0.04 to 0.25)	0.28 (0.16 to 0.43)	0.1 (0.02 to 0.23)	0.14 (0.05 to 0.28)	0.36 (0.25 to 0.51)	0.48 (0.32 to 0.67)	1.18 (0.91 to 1.5)	1.02 (0.8 to 1.27)	0.41 (0.24 to 0.63)
		**35 to 54**	0.04 (0 to 0.09)	0.08 (0.02 to 0.16)	0.17 (0.07 to 0.3)	0.11 (0.05 to 0.18)	0.02 (0 to 0.06)	0.35 (0.18 to 0.57)	0.56 (0.4 to 0.77)	1.07 (0.81 to 1.36)	1.27 (0.9 to 1.76)	0.57 (0.3 to 1.03)
		**55+**	0.01 (0 to 0.04)	0.03 (0 to 0.13)	0.08 (0.01 to 0.17)	0.07 (0.01 to 0.16)	0.07 (0.01 to 0.15)	0.1 (0.03 to 0.18)	0.19 (0.07 to 0.35)	0.82 (0.55 to 1.14)	0.57 (0.39 to 0.76)	0.59 (0.43 to 0.79)
	**iTaukei**	**1 to 4**	1.15 (0.84 to 1.49)	1.05 (0.63 to 1.62)	1.66 (1.32 to 2.09)	0.85 (0.61 to 1.1)	0.51 (0.33 to 0.72)	0.01 (0 to 0.05)	0	0	0.03 (0 to 0.1)	0
		**5 to 14**	0.63 (0.47 to 0.81)	3.2 (2.67 to 3.98)	1.25 (1.09 to 1.4)	1.28 (1.16 to 1.4)	0.33 (0.24 to 0.45)	0.01 (0 to 0.02)	0.03 (0 to 0.07)	0 (0 to 0.02)	0.01 (0 to 0.03)	0 (0 to 0.01)
		**15 to 34**	0.75 (0.64 to 0.87)	0.99 (0.86 to 1.13)	2.31 (2.05 to 2.58)	1.34 (1.14 to 1.56)	0.67 (0.54 to 0.83)	0 (0 to 0.01)	0.01 (0 to 0.01)	0.01 (0 to 0.02)	0.01 (0 to 0.03)	0
		**35 to 54**	0.47 (0.37 to 0.58)	1.19 (1.02 to 1.36)	1.42 (1.18 to 1.69)	1.54 (1.28 to 1.84)	0.59 (0.45 to 0.75)	0	0 (0 to 0.01)	0.02 (0 to 0.04)	0.01 (0 to 0.04)	0.01 (0 to 0.02)
		**55+**	0.48 (0.36 to 0.61)	0.84 (0.67 to 1.04)	1.08 (0.89 to 1.32)	0.92 (0.71 to 1.19)	0.91 (0.74 to 1.1)	0	0	0.01 (0 to 0.02)	0 (0 to 0.02)	0.01 (0 to 0.02)

iTaukei household sizes had a median of 5 residents and mean of 4.9 residents compared with a median of 4 residents and mean of 4.0 residents for non-iTaukei households. Data from lunchtime contacts indicated that contact rates did not only reflect household structure. Whilst eating dinner at home was almost universal (95%), 22% of iTaukei respondents had lunch contacts away from home, as did 21% of non-iTaukei. Those reporting lunch away from home also reported more contacts than those lunching at home (mean, median and IQR 8.0, 6, 3 to 9 and 4.5, 4, 2 to 6 contacts, respectively, *p*<0.0001). Participants eating lunch away from home (Table 3) had contact patterns indicating similar age and ethnically assortative mixing as seen in the overall contact pattern.

**Table 3:** Unweighted mean number of non-household lunch contact by age and ethnicity (bootstrap 95% confidence intervals).

		Contacts										
		iTaukei						Non-iTaukei				
		Age	0 to 4	5 to 14	15 to 34	35 to 54	55+	0 to 4	5 to 14	15 to 34	35 to 54	55+
**Participants**	**Non-iTaukei**	**1 to 4**	NA	NA	NA	NA	NA	NA	NA	NA	NA	NA
		**5 to 14**	0.08 (0 to 0.18)	0.7 (0.19 to 1.34)	0.19 (0.03 to 0.36)	0.43 (0.05 to 1.14)	0	0.14 (0.03 to 0.26)	1.78 (1.21 to 2.45)	0.78 (0.3 to 1.48)	0.84 (0.54 to 1.12)	0.38 (0.17 to 0.64)
		**15 to 34**	0.19 (0 to 0.67)	0.22 (0 to 0.69)	0.25 (0 to 0.67)	0.22 (0 to 0.78)	0.19 (0 to 0.67)	0.56 (0.24 to 1)	0.5 (0.09 to 1.06)	2.38 (1.68 to 3.21)	1.69 (1.08 to 2.46)	0.72 (0.22 to 1.5)
		**35 to 54**	0	0	0	0.05 (0 to 0.15)	0	1 (0.06 to 2.14)	1 (0.33 to 1.83)	1.95 (0.92 to 3.18)	3.3 (1.28 to 6)	2.1 (0.35 to 4.4)
		**55+**	0	0	0	0	0	0	0.5 (0 to 2)	0.75 (0 to 3)	1.25 (0 to 4)	0.25 (0 to 1)
	**iTaukei**	**1 to 4**	2 (0.33 to 4)	2.67 (0 to 8.59)	0.83 (0 to 1.67)	1.33 (0.33 to 2.33)	0.33 (0 to 0.8)	0	0	0	0	0
		**5 to 14**	0.65 (0.42 to 0.93)	4.86 (3.72 to 6.51)	1.3 (1.07 to 1.56)	1.27 (1.08 to 1.45)	0.38 (0.23 to 0.57)	0.01 (0 to 0.03)	0.04 (0 to 0.12)	0	0.01 (0 to 0.03)	0.01 (0 to 0.03)
		**15 to 34**	0.86 (0.54 to 1.24)	1.28 (0.96 to 1.68)	3.42 (2.79 to 4.11)	2.15 (1.56 to 2.95)	0.86 (0.45 to 1.48)	0	0	0.03 (0 to 0.07)	0.03 (0 to 0.06)	0
		**35 to 54**	0.54 (0.17 to 1)	1.37 (0.82 to 2.08)	2.44 (1.48 to 3.59)	2.76 (1.77 to 3.94)	0.93 (0.41 to 1.56)	0	0	0.05 (0 to 0.17)	0.05 (0 to 0.17)	0
		**55+**	1 (0.48 to 1.62)	1.39 (0.76 to 2.11)	1.93 (1.17 to 2.9)	1.79 (0.91 to 2.87)	1.32 (0.75 to 2)	0	0	0	0	0

**Fig 2:**
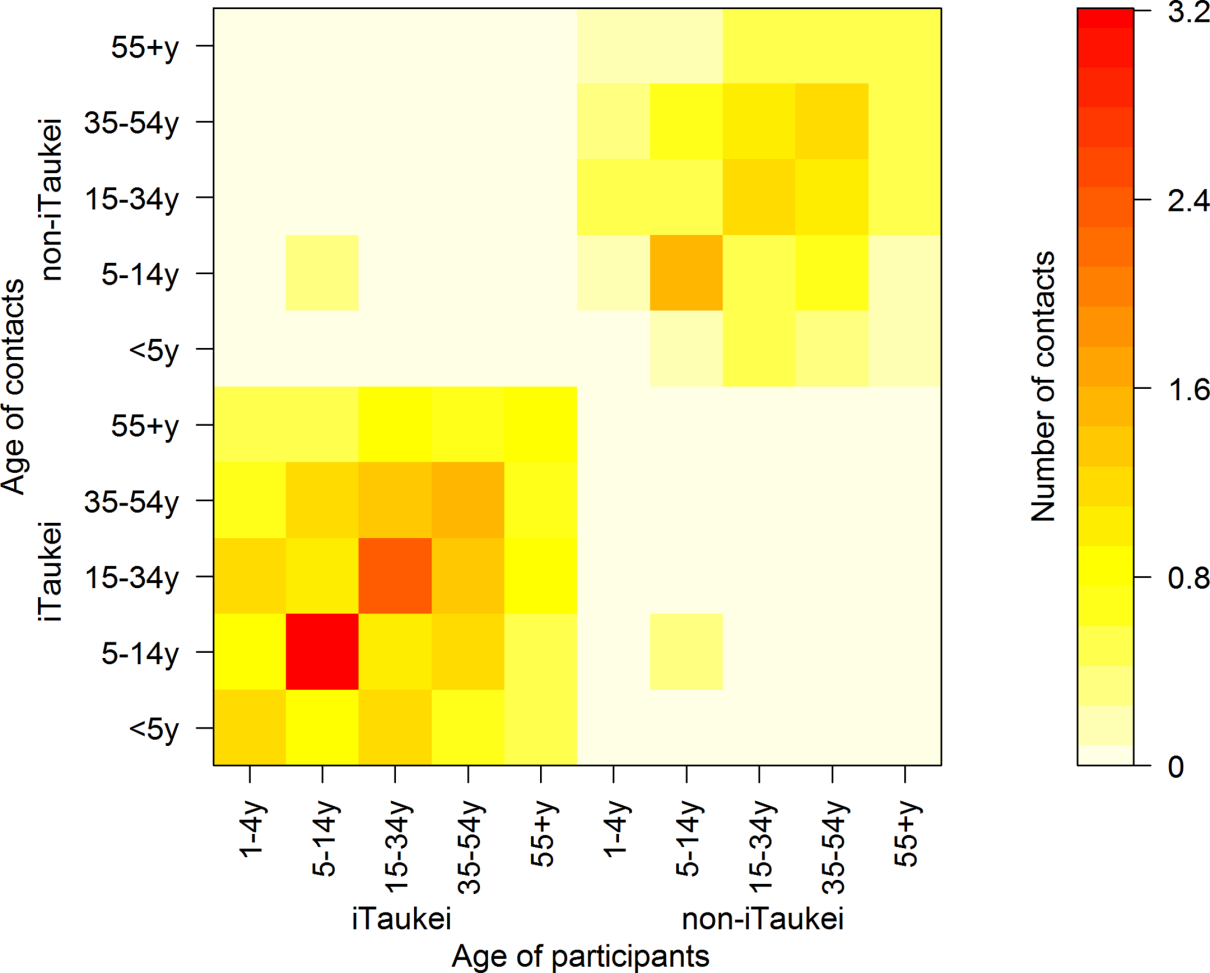
Age and ethnicity structured mixing matrices of reciprocity-weighted unique mealtime contacts per day.

After adjusting for reciprocity of contacts (census population and survey participant pyramids are shown in Supplementary S1 Fig), total daily contact data indicated sparse mixing between iTaukei and non-iTaukei ethnicity categories in all but school-age children (Fig 2). Age-assortative mixing was apparent within the two ethnicity categories, along with off-diagonal mixing, indicative of parent-child contact. In both ethnic categories, school-age children had the highest mean contact rates, followed by working-age adults. In contrast to the iTaukei pre-school children, the non-iTaukei children aged 1 to 4 years exhibited disassortative mixing, with more contacts reported within the working age adults than children of the same age.

We next examined the influence of unit increases in number of contacts on seropositivity for anti-Vi IgG *S*. Typhi amongst 1,559 participants (1,530 with complete data) from unvaccinated areas of Fiji. Age-adjusted seropositivity showed no correlation at threshold 64 EU used as a marker of any previous or current infection (odds ratio (OR) 1.01; 95% CI 0.99 to 1.02; *p* = 0.3) but some evidence of association at 100 EU, posited as indicative of recent infection (OR 1.02; 95% CI 1.00 to 1.03; *p* = 0.002) (Supplementary S3 File).

We performed further age-adjusted analyses to identify potential confounding variables and ascertain drivers for any such observed effect (Supplementary S3 File). Examination by ethnicity of contacts across both participant ethnic categories combined identified elevated odds ratios for association between unit increase in number of iTaukei contacts and the 100 EU recent infection serological threshold. No association was found for number of non-iTaukei contacts and seropositivity. The iTaukei contact rate association was not influenced by adjustment for participant ethnicity, the number of non-iTaukei contacts, or eating lunch outside the home when examined by multivariable regression (Supplementary S3 File). Analysis stratified by participant ethnicity found an effect of increasing contact rates in increasing seroprevalence amongst iTaukei participants but not non-iTaukei participants.

A parsimonious epidemiological model was constructed for serological association of recent infection with age-adjusted iTaukei contact rates in the unvaccinated iTaukei group, with non-iTaukei excluded from the final model due to the absence of observed association between any contact rates and seropositivity. Evidence of association was observed at the 100 EU threshold (“recent infection”) for a 1.026 (1.007 to 1.044) per contact increase in odds of seropositivity, after adjusting for age (Table 4).

**Table 4:** Logistic regression analysis of association between anti-Vi IgG seropositivity (100 EU), iTaukei daily contact number and participant age in 1,189 iTaukei participants from areas of Fiji never vaccinated against typhoid.

Variable	Odds ratio (95%CI)	*p*-value	AIC
iTaukei contact (per)	1.026 (1.007 to 1.044)	0.006	1046.3
Age (per year)	1.023 (1.014 to1.030)	<0.0001	

### Travel and internal migration patterns

**Fig 3:**
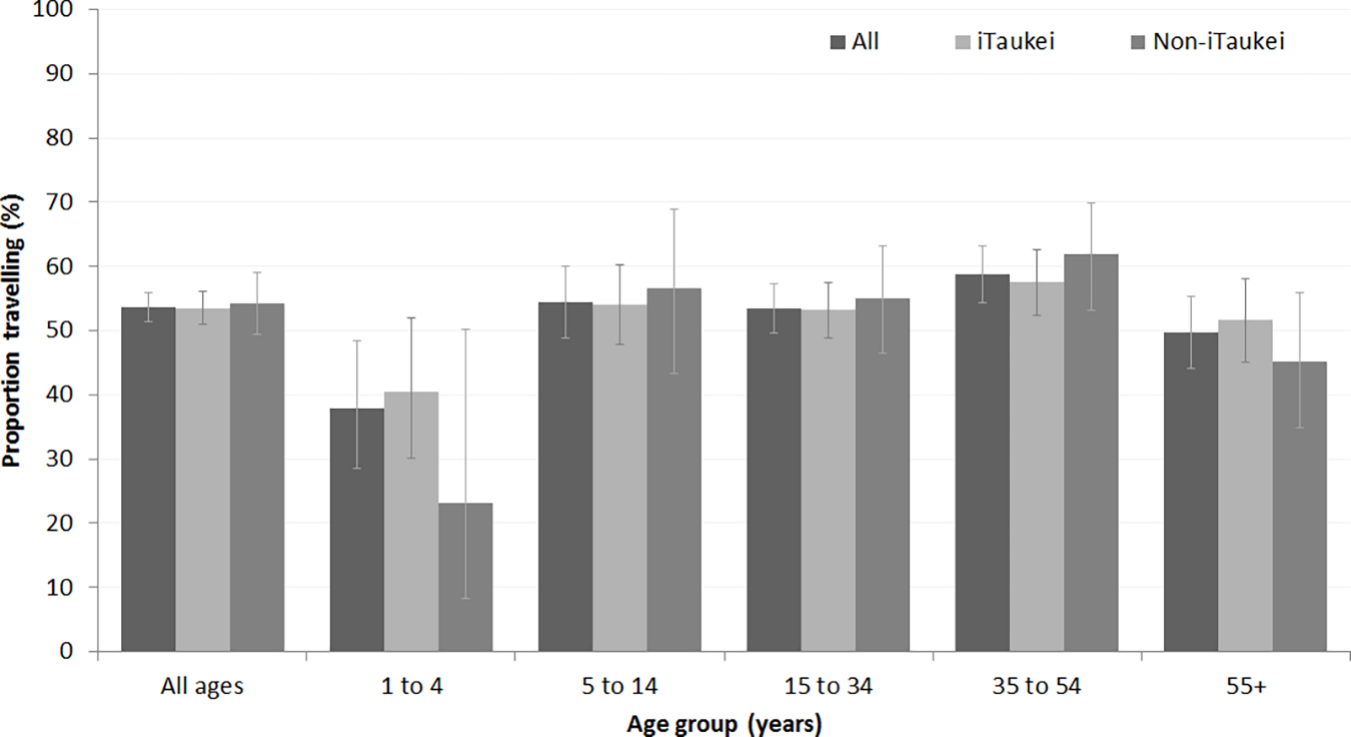
Travel outside of the community in the past week.

Local travel was common amongst the survey participants, with over half reporting travel outside of their residential community in the past week for all but the youngest and oldest age groups (Fig 3). Similar proportions of iTaukei and non-iTaukei reported travel in the past week. Recent travel was similar for urban, rural, and peri-urban residents (data not shown). Most travel was within the geographical administrative division (i.e. Central, Eastern, Northern, or Western Division of Fiji); of 974 participants reporting travel, 43 (4.2%, 95%CI 3.2% to 5.6%) reported travel to another Division in the past week.

Overall 37.2% (670/1803, 95% CI 35.0 to 39.4%) reported having moved residential community in their lifetimes. Participants aged 40 to 44 were most likely to have moved from a different place (Supplementary S2A Fig) to their current community of residence. Those aged 20 to 24 years were most likely to report having moved in the past 5 years, regardless of ethnicity category (Supplementary S2B and S2C Fig). Non-iTaukei children were more likely to have moved than iTaukei children, while adult iTaukei were more likely to have moved recently than non-iTaukei.

### Animal ownership and contact

Animal ownership was common in both ethnicity categories and across urban/peri-urban and rural households (Supplementary S1A and S1B Table). Pigs were the most commonly owned animal (28% of households (95% CI22 to 26%) despite being infrequently kept by non-iTaukei Fijians (5.7%, 95% CI 3.8 to 8.4%). Chickens were also widely kept, particularly by non-iTaukei (36%, 95% CI 31.5 to 40.8%). In rural communities, 20.8% (95% CI 18.4 to 23.4%) owned horses, with comparable ownership in both ethnic groups. This association contrasted with goat ownership, which was predominantly amongst rural non-iTaukei (30.8, 95%CI 24.4 to 38.1%, compared with mean prevalences <10% amongst other groups). Few Fijians reported keeping sheep (1.2% 95% CI 0.8 to 1.8%). Physical contact with wild rats or mongooses was reported infrequently (Supplementary S1C Table) despite sightings of these by 90% and 75% of participants, respectively.

## Discussion

Empirical research on contact patterns for infectious disease modelling has to date primarily considered epidemiological contacts for transmission of sexual or respiratory diseases. Social mixing patterns of direct relevance to enteric infections, or patterns of animal contact relevant to zoonotic spillover are less studied.

Using unique daily mealtime contacts, our social contact survey of Fiji found that within iTaukei and non-iTaukei ethnic groups there is age-assortative mixing, even within broad age categories, similar to contact patterns studied in the transmission of respiratory diseases seen in Asian or European settings [6,11–13]. We found minimal social mixing between people of the two ethnic categories, with inter-ethnic mixing most common amongst school-age children. iTaukei participants had higher mean daily contact rates than non-iTaukei participants. Examination of extra-domestic lunchtime contacts indicates that these patterns are replicated outside the home, showing that data do not simply reflect household structure. High levels of mobility in the population for all ages from 5 years upwards (overall >50% travelling in the previous week) suggest that communities on these larger Fijian islands are not isolated and transmission between urban and rural populations is readily feasible.

These data suggest it is plausible that effectively independent epidemics could occur in iTaukei and non-iTaukei residents of Fiji, for pathogens whose transmission can be approximated by mealtime contacts, given the low rates of substantive hetero-ethnic contact. The higher contact rates amongst iTaukei Fijians would more readily sustain person-to-person transmission than the rates in non-iTaukei Fijians. Analysis of age-adjusted contact rates and anti-Vi IgG to *Salmonella* Typhi found association with inter-iTaukei contacts and titres above a threshold that may be indicative of recent past infection but no association for contact rates involving non-iTaukei, further supporting use of these ethnically-structured social contact data in infectious disease modelling. Our recording of animal ownership by ethnicity enables estimation of the impact of differential seeding of zoonotic diseases such as avian influenza were they to first arrive in the Pacific as an epizootic.

The absolute contact rates obtained in this study cannot readily be compared with those from Mossong [6] and others which are primarily conversational in nature and not restricted to mealtimes. Nor do we attempt to document changing social contact patterns during acute illness [46,47]. However, their origins in mealtime contact do not limit these data to application in enteric disease only. As Goeyvaerts and colleagues [48] note, the importance of empirically-obtained social mixing rates is that they represent relative mixing patterns between population subgroups as proxies for the distribution of mechanisms of disease transmission. Melegaro and colleagues’ study of airborne viral pathogens [49] found “that intimate types of contacts explain the pattern of acquisition of serological markers by age better than other types of social contacts”.

In the absence of setting-specific data, these data might be very cautiously applied to use in other Pacific Island countries and territories, though more applicable to larger states than to low-lying small islands given that data collection excluded Fiji’s Eastern Division. Unadjusted iTaukei contact rates could be applied in many settings; unadjusted inter-ethnicity contact patterns could have potential application in settings such as French Polynesia where the estimated ethnically non-Polynesian population is relatively large at 22% [50], though do not account for the different social and cultural norms of such settings.

Our survey demonstrates that it is feasible and socially acceptable to gather data on social mixing not only by age but by ethnicity, in settings where heterogeneity may be of relevance to transmission networks and dynamics. Interestingly, we found non-iTaukei pre-school children had non-assortative mixing, in that they had greater contact with older age groups rather than with children of the same age, suggesting mealtime contact within a small family structure. Similar findings were reported in a UK study of under-ones [51]. This contrasts to assortative mixing in iTaukei pre-schoolers, consistent with sustained high birth rates/large extended families in iTaukei Fijians [39] and the divergent demographic trends in iTaukei and non-iTaukei Fijians (Supplementary S1 Fig). Compared with social contact survey settings overseas, low relative contact rates in older adult Fijians may reflect both lower adult life expectancy [52] as well as different social mixing patterns.

We found that non-iTaukei participants, predominantly comprising Indo-Fijians, were under-represented in the survey relative to census estimates, despite use of a structured sampling method. This may to some extent reflect continued outmigration and potentially within-country rural-to-urban migration differentially increasing nursing zones populations in majority Indo-Fijian areas above the numbers used in the sampling frames. Boosted surveys of non-iTaukei residents could address this, though value of the expected potential gains in precision would need consideration. The high reported enrolment rate likely reflects incomplete documentation of candidate participants approached who declined involvement.

Theoretical mixing structures that are not informed by data are largely being replaced in infectious disease modelling by contact patterns derived from data. Traditional, line-listing, prospective, paper-based, contact diaries can be demanding for participants and in data entry/analysis. Methods of measuring contact utilising portable electronic devices, such as mobile phone tracing [53,54] and RFID tagging [55,56] increasingly offer methods for collecting rich data on contact patterns but can involve substantive cost and/or complexity. There can be an advantageous degree of simplicity in asking people with whom they ate yesterday and where they travelled in the last week. Data for model parameterisation can be collected in a single-contact survey potentially alongside serology and behavioural or environmental risk data. Although retrospective survey responses may risk recall bias, we found that the previous day’s lunch and dinner partners were readily recallable by participants. This also reduces respondent fatigue and avoids potential for behaviour modifications that a prospective diary might trigger. Social response bias is reduced by reassurance that individual responses are kept confidential, and the socially-acceptable nature of enquiry, including careful structuring of ethnicity questions.

Whilst eating patterns themselves are an important public health topic with regards to the enormous impact of the epidemic of non-communicable disease in the Pacific and worldwide [57,58] they also offer insights for infectious disease epidemiologists and modellers. “Who-eats-with-whom” reflects social intimacy as well as specific food-borne and fomitic transmission risks and can effectively document ethnic- as well as age-assortative mixing. The universality of food-sharing as a human experience lends this approach, developed for enteric infectious diseases in Pacific Islands, to a range of settings where people interact and infections may transmit.

## Supporting information

S1 File Mealtime social contact questionnaire. A questionnaire data collection tool with integrated interview guide for mealtime social contacts by meal, setting, age and ethnicity.
(DOCX)

S2 File Supplementary data excel file. Sheet 1: line list of mealtime social contacts.
Sheet 2: line list of animal contacts.
Sheet 3: line list of travel in past week.
Sheet 4: line list of moving community.
(XLSX)

S3 File Seropositivity and social contact regression analysis. (PDF)

S1 Fig Age distribution (count) of iTaukei and non-iTaukei in Fiji in A) 2007 census and B) 2013 social contact survey.
(TIF)

S2 Fig Lifetime prevalence of having moved community and moved in the last five years for A) all participants, B) iTaukei participants, C) non-iTaukei participants, by five-year age bands. Hollow points denote 95% confidence intervals.
(TIF)

S1 Table Animal contact by ethnicity and geography (A: owned livestock, B: other owned domesticated animals, C: physical contact with wild rodents).
(DOCX)
