# Supplementary material for: Social mixing in Fiji: Who-eats-with-whom contact patterns and the implications of age and ethnic heterogeneity for disease dynamics in the Pacific Islands
Source: PLoS One. 2017 Dec 6;12(12):e0186911. doi: 10.1371/journal.pone.0186911 (PMC5718486; doi:10.1371/journal.pone.0186911)
Supplement: S1 File — A questionnaire data collection tool with integrated interview guide for mealtime social contacts by meal, setting, age and ethnicity. (DOCX) [file pone.0186911.s001.docx]

**I’d like to ask you about who you had meals with yesterday.** If asked, this is because typhoid may be spread on food.

| Q1a. First, where did you eat lunch yesterday?  Q1b. And where did you eat dinner yesterday? | | Lunch  (X one only) | | Dinner  (X one only) | Q2 |
| --- | --- | --- | --- | --- | --- |
| 1 | Home | | □ | □ | Ask: “how many people of each age also ate the same food?” |
| 2 | Somebody else’s home | | □ | □ |  |
| 3 | Buffet / lovo at church, village social gathering or similar | | □ | □ |  |
| 4 | Workplace/school – brought food from home/from somebody else’s home | | □ | □ |  |
| 5 | Workplace/school – cafeteria food | | □ | □ | Ask: “how many friends, family or colleagues of different ages did you share a table with?” |
| 6 | Restaurant/cafe/food court/takeaway (takeaway or ate there, includes hotel restaurants) | | □ | □ |  |
| 7 | Outdoor food stall (takeaway or ate there). | | □ | □ |  |
| 8 | Bought from a walking food seller | | □ | □ |  |
| 9 | Didn’t eat lunch/dinner | | □ | □ |  |
| 10 | Other:________________________ | | □ | □ |  |
| -1 | Don’t know | | □ | □ |  |
| -2 | Refuse to say | | □ | □ |  |

Q2. **I’d like you to think about how many people of different ages you shared food with, first at lunch, then at dinner. (**The participant should not include him or herself.)

(1) Food of the same source, where everybody who ate it can be recorded

**(1) For people who ate at home, somebody else’s home, at a buffet, or brought food to work,**

**ask “how many people of each age also ate the same food?”**

(food made in the same kitchen, shared at the meal, or from the same buffet)

**(2) For people who ate at a work cafeteria, from a roadside stall/street seller or**

**a restaurant/food court, ask “how many friends, family or colleagues of different ages did you share a table with?”**

(or equivalent to a table if ate outdoors)

(2) If the food was sold, and the person answering has no way of knowing how many other people ate it, ask instead about how many people they ate with.

Q3. **First lunch, starting with children under 5:**

**- of these people, how many were iTaukei Fijian, Indo-Fijian, or other?**

Q4. **Then dinner, starting with children under 5:**

**- of these people, how many were iTaukei Fijian, Indo-Fijian, or other?**

Q5. **And finally, how many of these people were the same at both lunch and dinner?**

**- of these people, how many were iTaukei Fijian, Indo-Fijian, or other?**

Go through each age group. Record exact numbers for numbers 0 through to 15. If the participant is concerned about getting it exactly right, tell them it is ok if they are a little bit out or can’t remember exactly.

If more than 15, use these bands: 16-24, 25-49, 50-99, 100+. Use also:-1=don’t know, -2 = refused, -3 not applicable.

| **Age:** | **0 to 4** | | | **5 to 14** | | | **15 to 34** | | | **35 to 54** | | | **55+** | | |
| --- | --- | --- | --- | --- | --- | --- | --- | --- | --- | --- | --- | --- | --- | --- | --- |
| **Q2(1)**  **Lunch** |  | It |  |  | It |  |  | It |  |  | It |  |  | It |  |
|  |  | Ind |  |  | Ind |  |  | Ind |  |  | Ind |  |  | Ind |  |
|  |  | Oth |  |  | Oth |  |  | Oth |  |  | Oth |  |  | Oth |  |
| **Q2(2) Dinner** |  | It |  |  | It |  |  | It |  |  | It |  |  | It |  |
|  |  | Ind |  |  | Ind |  |  | Ind |  |  | Ind |  |  | Ind |  |
|  |  | Oth |  |  | Oth |  |  | Oth |  |  | Oth |  |  | Oth |  |
| **Q5. Same people** |  | It |  |  | It |  |  | It |  |  | It |  |  | It |  |
|  |  | Ind |  |  | Ind |  |  | Ind |  |  | Ind |  |  | Ind |  |
|  |  | Oth |  |  | Oth |  |  | Oth |  |  | Oth |  |  | Oth |  |
