# Supplementary material for: Social mixing in Fiji: Who-eats-with-whom contact patterns and the implications of age and ethnic heterogeneity for disease dynamics in the Pacific Islands
Source: PLoS One. 2017 Dec 6;12(12):e0186911. doi: 10.1371/journal.pone.0186911 (PMC5718486; doi:10.1371/journal.pone.0186911)
Supplement: S3 File — (PDF) [file pone.0186911.s003.pdf]

Supplementary file S3 of **Social mixing in Fiji: who-eats-with-whom contact patterns and the implications of age and ethnic heterogeneity for disease dynamics in Pacific Islands.**

This supplementary document describes logistic regression analyses that were undertaken to develop understanding of association between mealtime social contact rates and seroprevalence in Fiji when taking into account potential confounding variables and ethnicity-based contact rates. These analyses follow an initial strong signal for association between total contact rates and seropositivity in the total survey group. These analyses are of residents of unvaccinated areas of mainland Fiji only, to reduce noise from vaccine-associated seropositivity on Taveuni island.

Figure S3 Fig 1 shows participants' contact rates, by participant ethnicity and proportion of contacts that were of iTaukei ethnicity. These data show that most participants have contact with only one ethnicity category of contact, which is usually the same category as the participant. There are a number of participants with mealtime contacts from the other ethnic category only, and relatively few with mealtime contacts across both ethnic categories.

S3 Fig 1. Number of contacts per iTaukei and non-iTaukei participant, by proportion of contacts that were iTaukei.

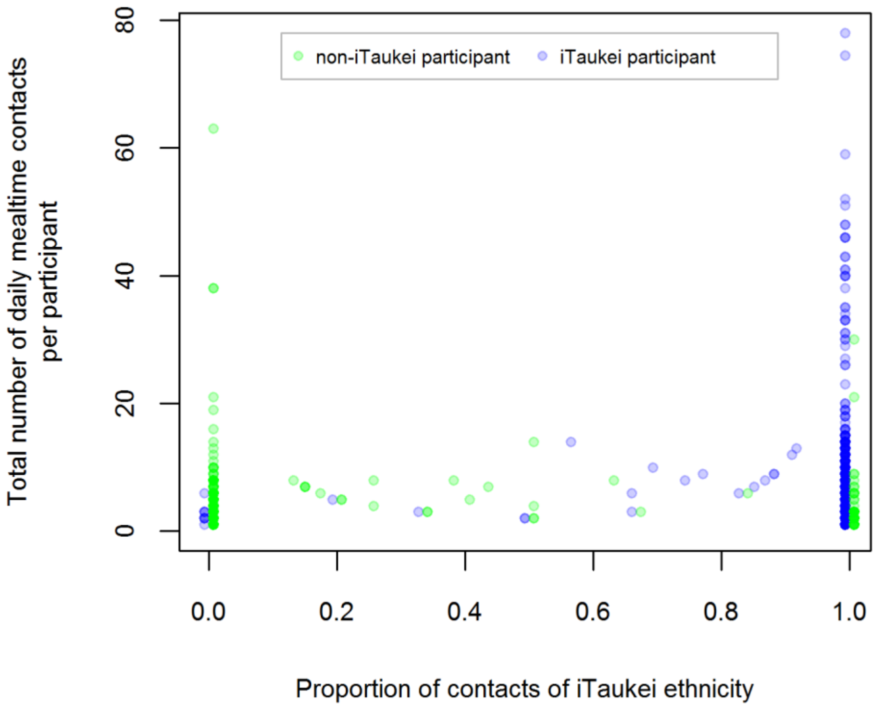

We first examined odds ratios for association between anti-Vi IgG seropositivity and total number of contacts, adjusting for participant age, without ethnicity adjustment for contacts or participants. This association was examined at both a seropositivity threshold of 64 EU, determined in prior research to be suggestive of previous or current infection, and 100 EU, more suggestive of recent or current infection. The recent infection 100 EU marker was considered to be more likely to be influenced by the participants' reported contact rates, as contact rates may have changed over time, particularly given the variation in contact rates demonstrated across age groups in the social mixing

survey. Data for 64 EU are reported here for completeness. Data were available for 1559 participants from unvaccinated areas of Fiji mainland.

Evidence of association between seropositivity and total contact rates (contacts of any ethnic category) was observed at 100 EU, with an effect size of approximately[1] 2% additional likelihood of seropositivity per additional contact. No association was demonstrated at 64 EU. (S3 Tables 1 and 2)

The data were then analysed by ethnicity of contacts (S3 Table 1 and 2). Some evidence was observed for association with iTaukei contacts at 64 EU, with stronger evidence of association at 100 EU, where data showed a similar effect size per contact to the above total contact rates analyses and greater strength of association. Weak association between increasing non-iTaukei contact rates and sero*negativity* was observed at 64 EU but not supported by examination at 100 EU. This suggested there was no association between non-iTaukei contact rates and seropositivity but that these data might contribute to non-differential misclassification when included in analysis of total contact rates.

S3 Table 1. 100 EU Vi IgG threshold, participants from unvaccinated areas, all ethnicities.

Model	Variable	Odds ratio	p-value	AIC
1.	Any contact<br>Age	1.02 (1.00 to 1.04)<br>1.025 (1.019 to 1.032)	0.02<br><0.0001	1376
2.	iTaukei contact<br>Age	1.025 (1.007 to 1.042)<br>1.026 (1.019 to1.033)	0.005<br><0.0001	1373.5
3.	Non-iTaukei contact<br>Age	0.96 (0.89 to 1.02)<br>1.025 (1.018 to 1.031)	0.2<br><0.0001	1378.9

S3 Table 2. 64 EU Vi IgG threshold, participants from unvaccinated areas, all ethnicities.

Model	Variable	Odds ratio	p-value	AIC
4.	Any contact<br>Age	1.01 (0.99 to 1.02)<br>1.026 (1.020 to 1.032)	0.33<br><0.0001	1854.1
5.	iTaukei contact<br>Age	1.01 (1.00 to 1.03)<br>1.026 (1.020 to 1.032)	0.08<br><0.0001	1852.1
6.	Non-iTaukei contact<br>Age	0.96 (0.91 to 1.00)<br>1.026 (1.020 to 1.031)	0.1<br><0.0001	1851.7

Analyses were next undertaken to determine whether an effect remained when data were stratified by the ethnicity of the survey participant. Amongst the 1,189 iTaukei participants, strong association with 100 EU seropositivity was observed for increasing total or iTaukei contact rates (S3 Table 3). This is consistent with the S3 Table 1 models and S3 Fig 1, as iTaukei participants comprise the large majority of respondents and primarily report iTaukei contacts. At 64 EU, models of iTaukei participants including total contacts or iTaukei contacts showed some association (S3 Table 4). Non-iTaukei contact rates in iTaukei participants showed weak evidence of association for 64 EU seropositivity and this association further weakened at 100 EU.

Amongst the 370 non-iTaukei participants, no association between seropositivity and contacts of any or either ethnic category was observed at 100 EU or 64 EU thresholds (S3 Tables 5 and 6).

S3 Table 3. 100 EU Vi IgG threshold, participants of iTaukei ethnicity from unvaccinated areas.

Model	Variable	Odds ratio (95% CI)	P value	AIC
7. Any contact, age adjusted	Any contact<br>Age (year)	1.026 (1.007 to 1.045)<br>1.023 (1.015 to 1.030)	0.005<br><0.0001	1046
8. Ethnically-stratified contact, age adjusted	iTaukei contact<br>non-iTaukei contact<br>Age (year)	1.026 (1.007 to 1.044)<br>1.24 (0.84 to 1.77)<br>1.023 (1.015 to 1.031)	0.005<br>0.2<br><0.0001	1047

S3 Table 4. 64 EU Vi IgG threshold, participants of iTaukei ethnicity from unvaccinated areas.

Model	Variable	Odds ratio (95% CI)	P value	AIC
9. Any contact, age adjusted	Any contact<br>Age (year)	1.01 (1.00 to 1.03)<br>1.026 (1.019 to 1.032)	0.1<br><0.0001	1416.7
10. Ethnically-stratified contact, age adjusted	iTaukei contact<br>non-iTaukei contact<br>Age (year)	1.01 (1.00 to 1.03)<br>1.40 (1.00 to 2.04)<br>1.026 (1.019 to 1.033)	0.1<br>0.06<br><0.0001	1415.1

S3 Table 5. 100 EU Vi IgG threshold, participants of non-iTaukei ethnicity from unvaccinated areas.

Model	Variable	Odds ratio (95% CI)	P value	AIC
11. Any contact, age adjusted	Any contact<br>Age (year)	0.95 (0.86 to 1.02)<br>1.035 (1.020 to 1.052)	0.3<br><0.0001	328.42
12. Ethnically-stratified contact, age adjusted	iTaukei contact<br>non-iTaukei contact<br>Age (year)	0.98 (0.80 to 1.10)<br>0.95 (0.85 to 1.02)<br>1.035 (1.020 to 1.052)	0.8<br>0.2<br><0.0001	330.28

S3 Table 6. 64 EU Vi IgG threshold, participants of non-iTaukei ethnicity from unvaccinated areas.

Model	Variable	Odds ratio (95% CI)	P value	AIC
13. Any contact, age adjusted	Any contact<br>Age (year)	0.95 (0.88 to 1.01)<br>1.027 (1.015 to 1.041)	0.1<br><0.0001	436.05
14. Ethnically-stratified contact, age adjusted	iTaukei contact<br>non-iTaukei contact<br>Age (year)	0.92 (0.75 to 1.04)<br>0.95 (0.88 to 1.01)<br>1.027 (1.015 to 1.040)	0.3<br>0.2<br><0.0001	437.83

Having observed an age-adjusted association between increasing daily number of iTaukei contacts and recent *Salmonella* Typhi infection, driven by contacts made by iTaukei participants, we examined the full participant dataset for the influence of covariates such as lunching away from home on this association (S3 Tables 7 and 8). As these covariates did not substantially influence the effect size or evidence of association, the model presented in the main paper is the parsimonious model accounting for iTaukei contacts and participant age only. As non-iTaukei participants showed no association between contact rates and seropositivity, the model shown in the main paper is that applying to iTaukei only.

S3 Table 7. 100 EU Vi IgG threshold multivariable regression models, unvaccinated areas, participants of all ethnicities

Model	Variable	Odds ratio (95% CI)	P value	AIC
15.	iTaukei contact	1.023 (1.005 to 1.041)	0.01	1374.8
	non-iTaukei contact	0.98 (0.91 to 1.03)	0.4	
	Age (year)	1.026 (1.019 to 1.033)	<0.0001	
16.	iTaukei contact	1.025 (1.006 to 1.044)	0.007	1376
	non-iTaukei contact	0.96 (0.87 to 1.02)	0.3	
	Age (year)	1.025 (1.018 to 1.032)	<0.0001	
	Non-iTaukei ethnicity	1.2 (0.8 to 1.8)	0.4	
17.	iTaukei contact	1.01 (1.00 to 1.04)	0.022	1375.4
	non-iTaukei contact	0.97 (0.91 to 1.02)	0.4	
	Age (year)	1.026 (1.019 to 1.034)	<0.0001	
	Lunch away from home	1.2 (0.8 to 1.6)	0.3	
18.	iTaukei contact	1.023 (1.004 to 1.042)	0.015	1376.5
	non-iTaukei contact	0.95 (0.87 to 1.02)	0.3	
	Age (year)	1.026 (1.019 to 1.033)	<0.0001	
	Non-iTaukei ethnicity	1.2 (0.8 to 1.8)	0.4	
	Lunch away from home	1.2 (0.8 to 1.7)	0.3	

S3 Table 8. 64 EU Vi IgG threshold multivariable regression models, unvaccinated areas, participants of all ethnicities

Model	Variable	Odds ratio (95% CI)	P value	AIC
19.	iTaukei contact<br>non-iTaukei contact<br>Age (year)	1.01 (0.99 to 1.03)<br>0.97 (0.91 to 1.01)<br>1.026 (1.020 to 1.032)	0.17<br>0.17<br><0.0001	1851.8
20.	iTaukei contact<br>non-iTaukei contact<br>Age (year)<br>Non-iTaukei ethnicity	1.01 (0.99 to 1.03)<br>0.97 (0.91 to 1.02)<br>1.026 (1.020 to 1.032)<br>0.92 (0.69 to 1.29)	0.2<br>0.3<br><0.0001<br>0.6	1853.6
21.	iTaukei contact<br>non-iTaukei contact<br>Age (year)<br>Non-iTaukei ethnicity<br>Lunch away from home	1.01 (1.00 to 1.03)<br>0.97 (0.91 to 1.02)<br>1.026 (1.021 to 1.033)<br>0.93 (0.67 to 1.3)<br>1.04 (0.78 to 1.37)	0.2<br>0.3<br><0.0001<br>0.7<br>0.8	1852.5
22.	iTaukei contact<br>non-iTaukei contact<br>Age (year)<br>Non-iTaukei ethnicity<br>Lunch away from home<br>Interaction for contacts	1.02 (0.99 to 1.03)<br>0.97 (0.91 to 1.02)<br>1.026 (1.021 to 1.033)<br>0.93 (0.67 to 1.3)<br>1.04 (0.78 to 1.37)<br>1.03 1.00 (0.94 to 1.05)	0.2<br>0.3<br><0.0001<br>0.7<br>0.8<br>1	1854.4

Reference

1. Cummings P. The relative merits of risk ratios and odds ratios. *Arch Pediatr Adolesc Med*. 2009;163(5):438-445.
