# Supplementary figures and images for: Social mixing in Fiji: Who-eats-with-whom contact patterns and the implications of age and ethnic heterogeneity for disease dynamics in the Pacific Islands
Source: PLoS One. 2017 Dec 6;12(12):e0186911. doi: 10.1371/journal.pone.0186911 (PMC5718486; doi:10.1371/journal.pone.0186911)

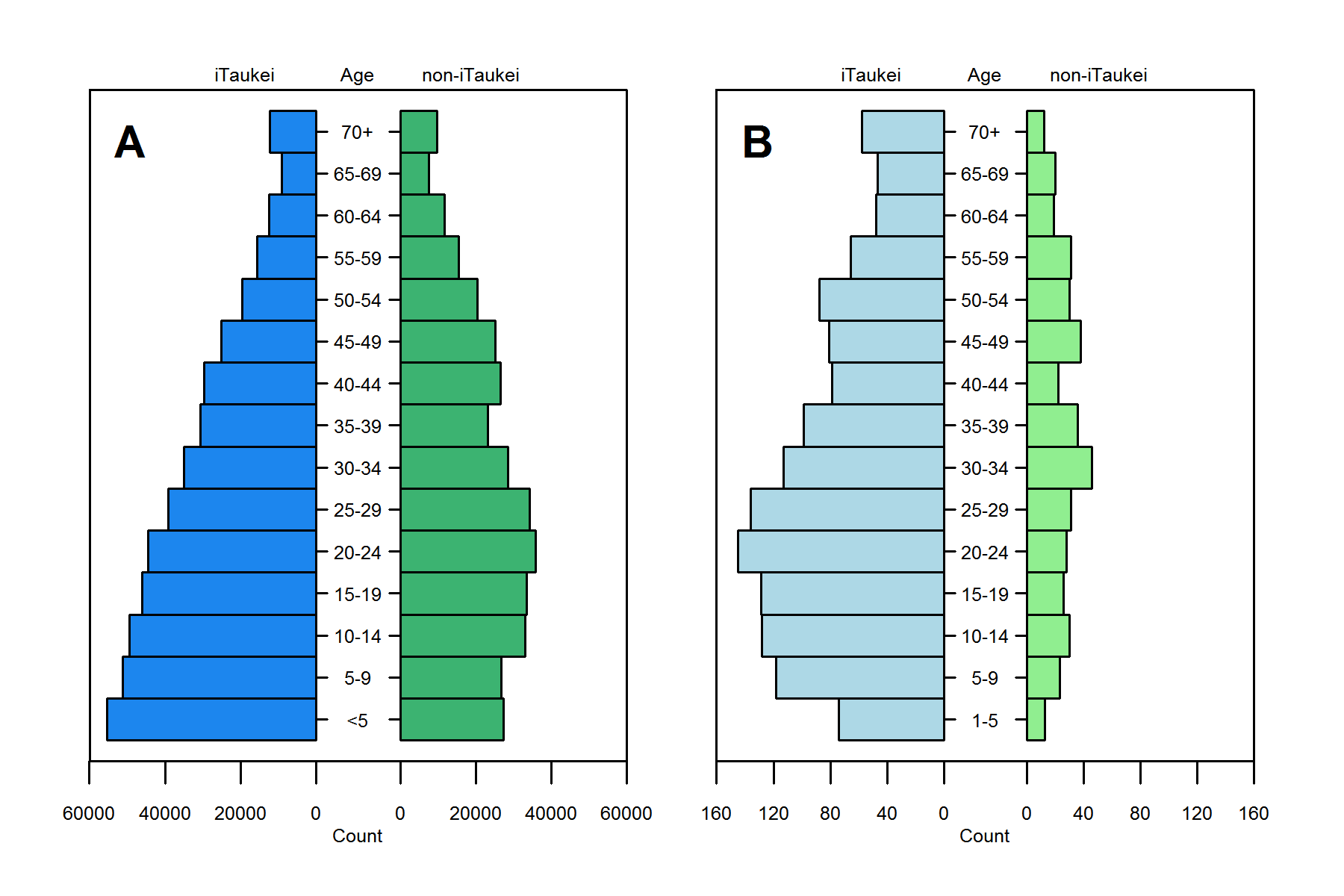

Supplement: S1 Fig — Age distribution (count) of iTaukei and non-iTaukei in Fiji in A) 2007 census and B) 2013 social contact survey. (TIF) [file pone.0186911.s004.tif]

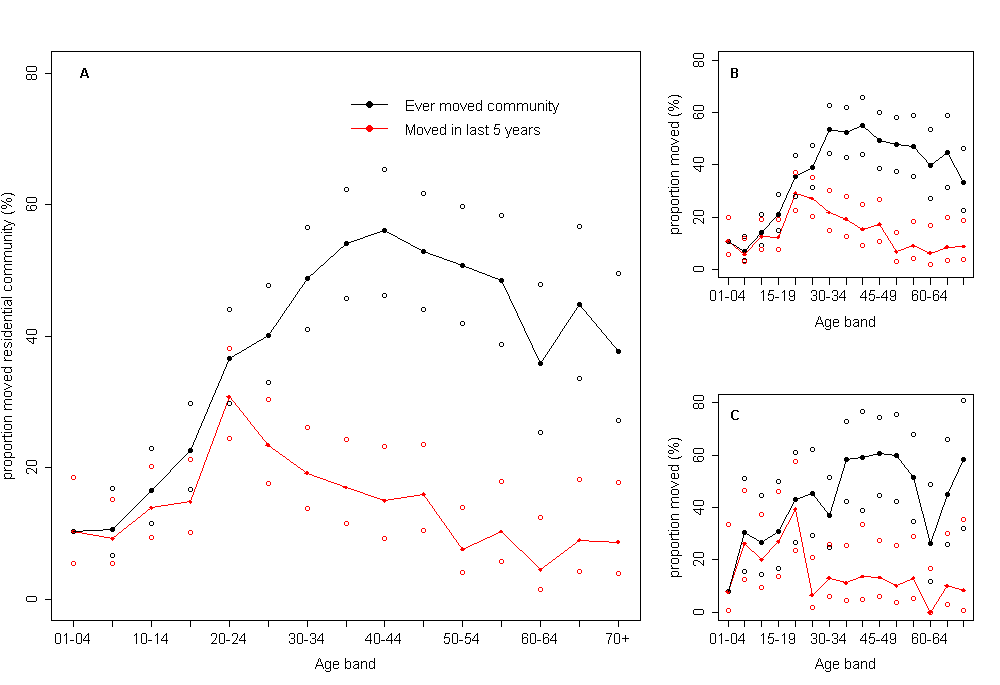

Supplement: S2 Fig — Lifetime prevalence of having moved community and moved in the last five years for A) all participants, B) iTaukei participants, C) non-iTaukei participants, by five-year age bands. Hollow points denote 95% confidence intervals. (TIF) [file pone.0186911.s005.tif]
