# Supplementary material for: Social mixing in Fiji: Who-eats-with-whom contact patterns and the implications of age and ethnic heterogeneity for disease dynamics in the Pacific Islands
Source: PLoS One. 2017 Dec 6;12(12):e0186911. doi: 10.1371/journal.pone.0186911 (PMC5718486; doi:10.1371/journal.pone.0186911)
Supplement: S1 Table — Animal contact by ethnicity and geography (A: owned livestock, B: other owned domesticated animals, C: physical contact with wild rodents). (DOCX) [file pone.0186911.s006.docx]

| A | Geography | Overall  n % (95% CI) | | iTaukei  n % (95% CI) | | Non-iTaukei  n % (95% CI) | |
| --- | --- | --- | --- | --- | --- | --- | --- |
| Chicken | All | 441 | 24.3 (22.4 to 26.3) | 295 | 20.9 (18.9 to 23.1) | 146 | 36 (31.5 to 40.8) |
|  | Urban | 23 | 4.6 (3.1 to 6.7) | 16 | 4.1 (2.6 to 6.6) | 7 | 5.9 (2.9 to 11.6) |
|  | Peri-urban | 55 | 19 (14.9 to 24) | 24 | 13.7 (9.4 to 19.6) | 31 | 27.2 (19.9 to 36) |
|  | Rural | 363 | 35.6 (32.7 to 38.6) | 255 | 30.1 (27.1 to 33.2) | 108 | 62.8 (55.4 to 69.7) |
| Cows | All | 388 | 21.4 (19.6 to 23.3) | 316 | 22.4 (20.3 to 24.7) | 72 | 17.8 (14.4 to 21.8) |
|  | Urban | 44 | 8.7 (6.6 to 11.5) | 42 | 10.9 (8.2 to 14.4) | 2 | 1.7 (0.5 to 5.9) |
|  | Peri-urban | 41 | 14.2 (10.6 to 18.7) | 27 | 15.4 (10.8 to 21.5) | 14 | 12.3 (7.5 to 19.6) |
|  | Rural | 303 | 29.7 (27 to 32.6) | 247 | 29.1 (26.2 to 32.3) | 56 | 32.6 (26 to 39.9) |
| Goats | All | 155 | 8.5 (7.3 to 9.9) | 91 | 6.5 (5.3 to 7.9) | 64 | 15.8 (12.6 to 19.7) |
|  | Urban | 29 | 5.7 (4 to 8.1) | 27 | 7 (4.9 to 10) | 2 | 1.7 (0.5 to 5.9) |
|  | Peri-urban | 26 | 9 (6.2 to 12.9) | 17 | 9.7 (6.2 to 15) | 9 | 7.9 (4.2 to 14.3) |
|  | Rural | 100 | 9.8 (8.1 to 11.8) | 47 | 5.5 (4.2 to 7.3) | 53 | 30.8 (24.4 to 38.1) |
| Pigs | All | 508 | 28 (26 to 30.1) | 485 | 34.4 (32 to 36.9) | 23 | 5.7 (3.8 to 8.4) |
|  | Urban | 64 | 12.7 (10.1 to 15.9) | 62 | 16.1 (12.7 to 20.1) | 2 | 1.7 (0.5 to 5.9) |
|  | Peri-urban | 57 | 19.7 (15.5 to 24.7) | 47 | 26.9 (20.8 to 33.9) | 10 | 8.8 (4.8 to 15.4) |
|  | Rural | 387 | 37.9 (35 to 41) | 376 | 44.3 (41 to 47.7) | 11 | 6.4 (3.6 to 11.1) |
| Sheep | All | 21 | 1.2 (0.8 to 1.8) | 14 | 1 (0.6 to 1.7) | 7 | 1.7 (0.8 to 3.5) |
|  | Urban | 0 | 0 (0 to 0.8) | 0 | 0 (0 to 1) | 0 | 0 (0 to 3.1) |
|  | Peri-urban | 11 | 3.8 (2.1 to 6.7) | 10 | 5.7 (3.1 to 10.2) | 1 | 0.9 (0 to 4.8) |
|  | Rural | 10 | 1 (0.5 to 1.8) | 4 | 0.5 (0.2 to 1.2) | 6 | 3.5 (1.6 to 7.4) |

| B | Geography | Overall  n % (95% CI) | | iTaukei  n % (95% CI) | | Non-iTaukei  n % (95% CI) | |
| --- | --- | --- | --- | --- | --- | --- | --- |
| Cats | All | 320 | 17.6 (16 to 19.5) | 219 | 15.5 (13.7 to 17.5) | 101 | 24.9 (21 to 29.4) |
|  | Urban | 84 | 16.6 (13.6 to 20.1) | 59 | 15.3 (12 to 19.2) | 25 | 21 (14.7 to 29.2) |
|  | Peri-urban | 63 | 21.8 (17.4 to 26.9) | 38 | 21.7 (16.2 to 28.4) | 25 | 21.9 (15.3 to 30.4) |
|  | Rural | 173 | 17 (14.8 to 19.4) | 122 | 14.4 (12.2 to 16.9) | 51 | 29.7 (23.3 to 36.9) |
| Dogs | All | 577 | 31.8 (29.7 to 34) | 397 | 28.2 (25.9 to 30.6) | 180 | 44.4 (39.7 to 49.3) |
|  | Urban | 146 | 28.9 (25.1 to 33) | 101 | 26.2 (22 to 30.8) | 45 | 37.8 (29.6 to 46.8) |
|  | Peri-urban | 98 | 33.9 (28.7 to 39.5) | 48 | 27.4 (21.4 to 34.5) | 50 | 43.9 (35.1 to 53) |
|  | Rural | 333 | 32.6 (29.8 to 35.6) | 248 | 29.2 (26.3 to 32.4) | 85 | 49.4 (42 to 56.8) |
| Horses | All | 265 | 14.6 (13.1 to 16.3) | 233 | 16.5 (14.7 to 18.6) | 32 | 7.9 (5.7 to 10.9) |
|  | Urban | 35 | 6.9 (5 to 9.5) | 33 | 8.5 (6.2 to 11.8) | 2 | 1.7 (0.5 to 5.9) |
|  | Peri-urban | 18 | 6.2 (4 to 9.6) | 18 | 10.3 (6.6 to 15.7) | 0 | 0 (0 to 3.3) |
|  | Rural | 212 | 20.8 (18.4 to 23.4) | 182 | 21.5 (18.8 to 24.4) | 30 | 17.4 (12.5 to 23.8) |

| C | Geography | Overall  n % (95% CI) | | iTaukei  n % (95% CI) | | Non-iTaukei  n % (95% CI) | |
| --- | --- | --- | --- | --- | --- | --- | --- |
| Mongooses | All | 110 | 6.1 (5.1 to 7.3) | 103 | 7.3 (6.1 to 8.8) | 7 | 1.7 (0.8 to 3.5) |
|  | Urban | 19 | 3.8 (2.4 to 5.8) | 18 | 4.7 (3 to 7.3) | 1 | 0.8 (0 to 4.6) |
|  | Peri-urban | 13 | 4.5 (2.6 to 7.5) | 9 | 5.1 (2.7 to 9.5) | 4 | 3.5 (1.4 to 8.7) |
|  | Rural | 78 | 7.6 (6.2 to 9.4) | 76 | 9 (7.2 to 11.1) | 2 | 1.2 (0.3 to 4.1) |
| Rats | All | 247 | 13.6 (12.1 to 15.3) | 220 | 15.6 (13.8 to 17.6) | 27 | 6.7 (4.6 to 9.5) |
|  | Urban | 56 | 11.1 (8.6 to 14.1) | 50 | 13 (10 to 16.7) | 6 | 5 (2.3 to 10.6) |
|  | Peri-urban | 38 | 13.1 (9.7 to 17.5) | 26 | 14.9 (10.3 to 20.9) | 12 | 10.5 (6.1 to 17.5) |
|  | Rural | 153 | 15 (12.9 to 17.3) | 144 | 17 (14.6 to 19.7) | 9 | 5.2 (2.8 to 9.6) |
